# Correction to “Nanoparticles (NPs)‐Meditated LncRNA AFAP1‐AS1 Silencing to Block Wnt/β‐Catenin Signaling Pathway for Synergistic Reversal of Radioresistance and Effective Cancer Radiotherapy”

**DOI:** 10.1002/advs.76442

**Published:** 2026-07-03

Zhuofei Bi, Qingjian Li, Xiaoxiao Dinglin, Ying Xu, Kaiyun You, Huangming Hong, Qian Hu, Wei Zhang, Chenchen Li, Yujie Tan, Ning Xie, Wei Ren, Chuping Li, Yimin Liu, Hai Hu, Xiaoding Xu, Herui Yao, "Nanoparticles (NPs)‐Meditated LncRNA AFAP1‐AS1 Silencing to Block Wnt/β‐Catenin Signaling Pathway for Synergistic Reversal of Radioresistance and Effective Cancer Radiotherapy," *Advance Science* 7 no. 18 (2020): 2000915, https://doi.org/10.1002/advs.202000915.

The authors regret to report that two errors were published in Figures 2G and 5A. The images of Liop2k/si‐1 group (row 1, column 2) and Liop2k/siCTL group (row 2, column 1) in Figure 2G, and NPs(siCTL) group (row 2, column 2) and Liop2k/si‐1 group (row 2, column 3) in Figure 5A were wrongly used during layout. The corrected figures are shown below in full, along with the original figure captions. These changes do not affect the interpretation of data or the conclusion of the study.

We apologize for this error.

**FIGURE 2:**
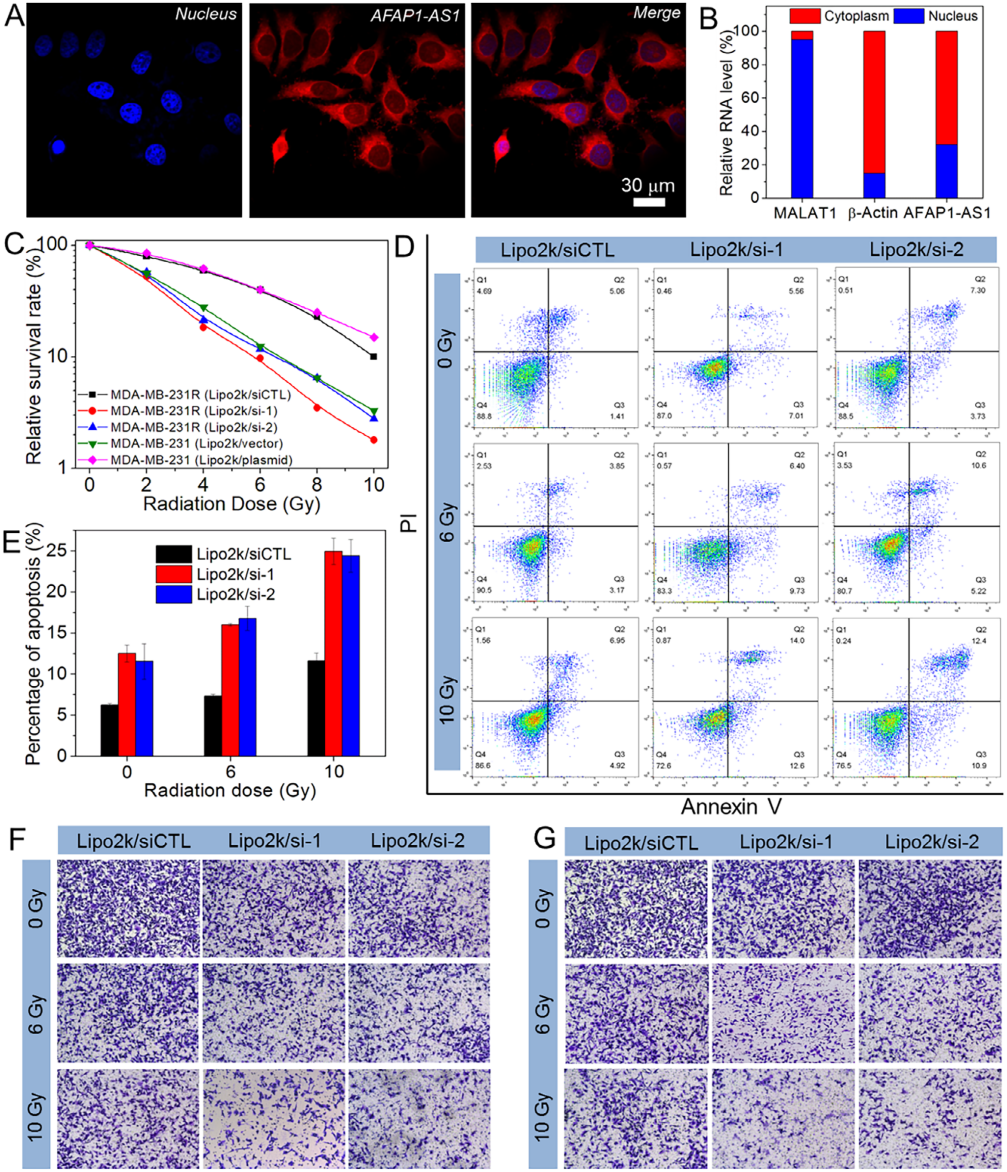
LncAFAP1‐AS1 is predominantly located in the cytoplasm, and its high expression promotes TNBC cell proliferation, migration, and invasion. (A) FISH images of MDA‐MB‐231R cells observed under CLSM. (B) LncAFAP1‐AS1 expression in the cytoplasm and nuclei of MDA‐MB‐231R cells determined by nuclear/cytoplasm fractionation. (C) Relative survival rate of MDA‐MB‐231 cells treated with the Lipo2k/plasmid complexes at a plasmid concentration of 1.5 µg/mL, followed by different doses of X‐ray radiation, or MDA‐MB‐231R cells treated with the Lipo2k/si‐1, Lipo2k/si‐2, or Lipo2k/siCTL complexes at a siRNA concentration of 30 nM, followed by different doses of X‐ray radiation. (D‐G) Flow cytometry profile (D) and percentage of apoptosis (E) of MDA‐MB‐231R cells treated with the formulas in (C), followed by different doses of X‐ray radiation. (F, G) Migration (F) and invasion (G) of MDA‐MB‐231R cells treated with the Lipo2k/si‐1 or Lipo2k/si‐2 complexes at a siRNA dose of 15 nM, followed by different doses of X‐ray radiation.

**FIGURE 5:**
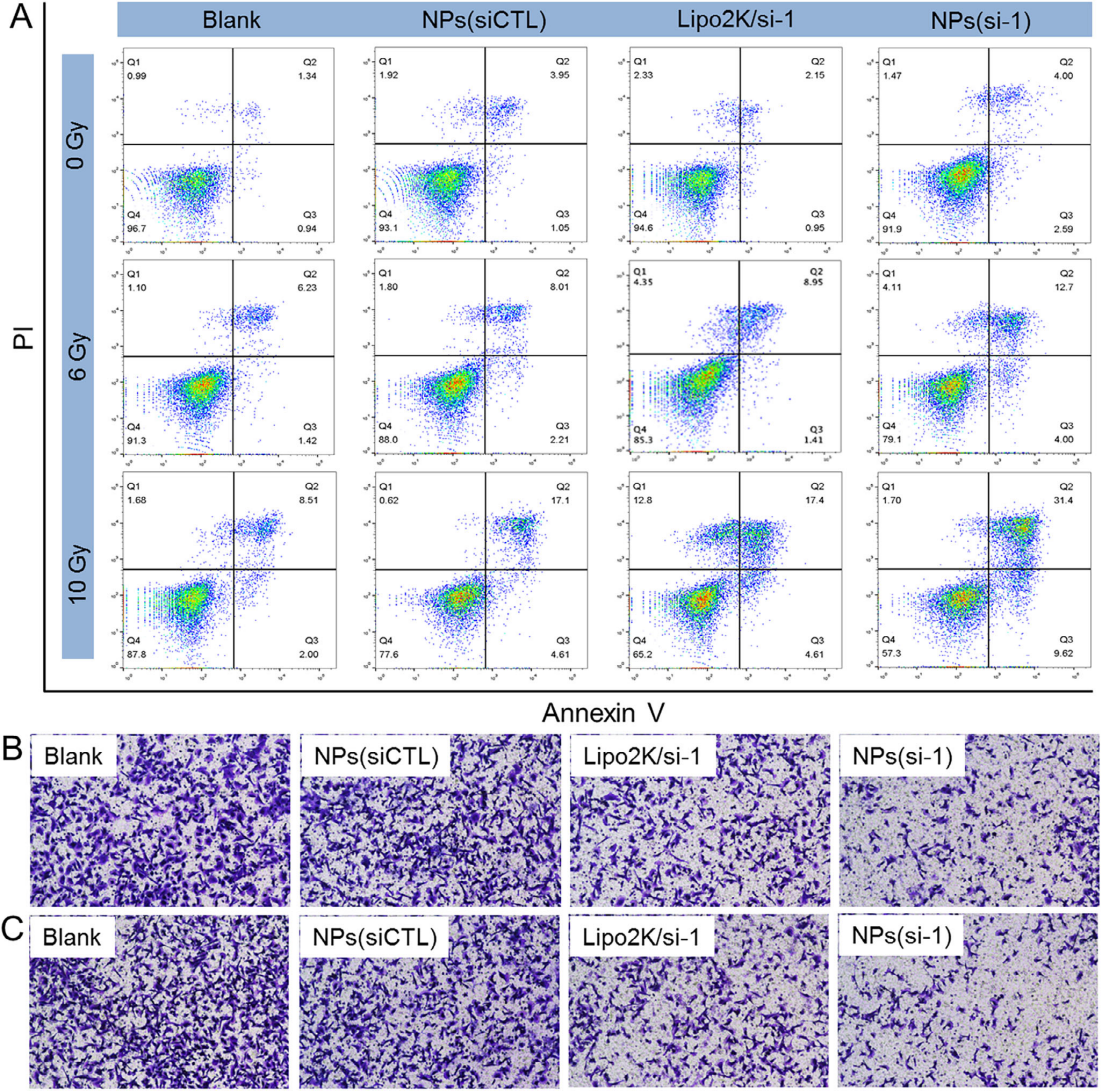
Reduction‐responsive NPs‐mediated lncAFAP1‐AS1 silencing enhances TNBC radiosensitivity and inhibits TNBC cell proliferation, migration, and invasion. (A) Flow cytometry profiles of MDA‐MB‐231R cells treated with the NPs(siCTL), Lipo2k/si‐1 complexes, or NPs(si‐1) at a siRNA concentration of 30 nM, followed by different doses of X‐ray radiation. (B, C) Migration (B) and invasion (C) of MDA‐MB‐231R cells treated with the formulas in (A) at a siRNA concentration of 30 nM, followed by 10 Gy of X‐ray radiation.

